# Superconducting PdTe Thin Film via Topotactic Transformation, toward Topological Superconductors

**DOI:** 10.1021/acsanm.6c00907

**Published:** 2026-05-29

**Authors:** Hee Taek Yi, Min Ge, Renjie Xie, Colby J. Stoddard, David H. Yi, Xiaoyu Yuan, Xiong Yao, Seongshik Oh

**Affiliations:** † Department of Physics & Astronomy, Rutgers University, Piscataway, New Jersey 08854, United States; ‡ The Instruments Center for Physical Science, University of Science and Technology of China, Hefei 230026, China; § Ningbo Institute of Materials Technology and Engineering, Chinese Academy of Sciences, Ningbo 315201, China; ∥ Department of Physics, Mellon College of Science, Carnegie Mellon University, Pittsburgh, Pennsylvania 15213, United States

**Keywords:** : PdTe thin films, Topological superconductivity, Majorana zero modes, Topotactic transformation, Molecular beam epitaxy

## Abstract

Topological superconductors (TSCs) hosting Majorana zero modes (MZMs) offer a pathway to fault-tolerant quantum computation. PdTe is a promising TSC candidate due to its topological surface states and a reasonable superconducting critical temperature of ∼4.5 K. However, it has been challenging to grow PdTe thin films with bulk-like superconducting properties. Here, we show that high-quality, superconducting PdTe thin films can be grown using molecular beam epitaxy (MBE). The films exhibit a sharp superconducting transition (*T*_onset_ = 4.43 K with transition width of 0.06 K), comparable to that of bulk crystals. This was made possible via a topotactic transformation from a PdTe_2_ buffer layer to a PdTe phase by growing Pd on top under Te-deficient conditions. Structural and transport analyses confirm the NiAs-type structure of PdTe as well as its two-dimensional superconducting behavior and excellent air stability. These findings suggest that the MBE-grown PdTe films and their heterostructures are a promising platform for topological superconductivity and Majorana physics.

## Graphical abstract

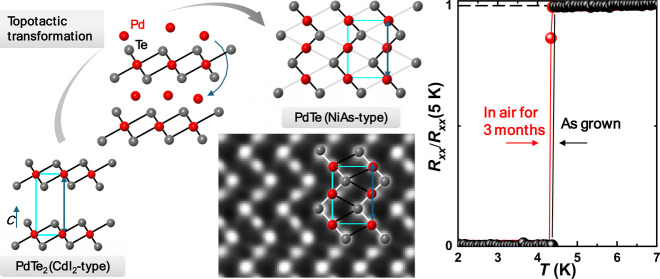

## Introduction

Topological superconductors (TSC), which host Majorana zero modes (MZM), have emerged as a promising platform for fault-tolerant computation relying on their non-Abelian statistics.^1–3^ TSCs can be realized by combining a conventional superconductor with a topological surface state, such as topological insulators, Weyl/Dirac semimetals with Fermi arcs, or magnetic materials. There have been three pathways toward achieving TSC states: a) search for intrinsic topological superconductors such as Fe­(Te,Se), b) engineering the proximity effect that combines superconductors (SC) with topological surface states in a heterostructure form, and c) utilizing the magnetic proximity effect in an interface between an s-wave SC and magnetic materials to create triplet superconductivity.^4–12^

PdTe has recently emerged as a promising new candidate for an intrinsic TSC. It possesses two essential ingredients for TSCs: superconductivity and topological surface states. The transition temperature of PdTe at 4.5 K is significantly higher than that of PdTe_2_. Furthermore, PdTe exhibits Dirac semimetal features with a bulk Dirac cone and topological surface states with Fermi arcs. The absence of strongly correlated 3d elements also suggests that the pairing mechanism is likely to be conventional electron–phonon coupling rather than exotic mechanisms such as spin fluctuations.^13–17^ Angle-resolved photoemission spectroscopy (ARPES) measurements on PdTe single crystals have revealed the coexistence of a fully gapped superconducting state on the surface and a gapless nodal state in the bulk. Specific heat measurements support this coexistence. Meanwhile, thermal conductivity measurements point toward multiple nodeless gaps.^13–16^ PdTe also offers significant advantages for TSC via the proximity effect in heterostructures. Its hexagonal crystal structure, with an in-plane lattice constant of 0.415 nm, closely matches those of a topological insulator, Bi_2_Se_3_, and an altermagnet, MnTe.^18–21^ Accordingly, high-quality heterostructures with a clean interface between PdTe and these related materials can be envisioned toward proximity-induced TSCs. All these properties make PdTe an attractive candidate for realizing TSCs, either intrinsically or in heterostructures. However, it has been challenging to grow high-quality superconducting PdTe thin films,^22–24^ with bulk-like *T*_c_ reported only recently on vacuum-annealed PLD (Pulsed Laser Deposition)-grown films.^25^

In this study, we report the synthesis of high-quality PdTe thin films, using MBE (Molecular Beam Epitaxy) technique. Transport measurements exhibit a high onset temperature (*T*_onset_ = 4.43 K) with *T*_0_ = 4.37 K and a residual resistivity ratio (*RRR*) of 10, consistent with the reported *T*_c_ of bulk crystals.^14–16^ We successfully achieved the PdTe phase by depositing Pd in Te-deficient conditions on a PdTe_2_ buffer layer via a topotactic transformation, a structural phase change mediated by atomic rearrangement.^26–29^ Comprehensive characterizations, including in situ Reflection High Energy Electron Diffraction (RHEED), X-ray Diffraction (XRD), low-temperature electric transport measurements, and Scanning Transmission Electron Microscopy (STEM), confirm the high crystallinity with *T*_c_ continuously tunable between those of PdTe_2_ and PdTe. These films also allowed the first Scanning Tunneling Microscopy (STM) studies on the PdTe system, answering key unresolved questions in this system, which will be reported in a follow-up paper.

## Results and Discussion

We grew PdTe thin films in a custom-built MBE system with a base pressure of ∼10^–10^ Torr, on 10 × 10 × 0.5 mm^3^ Al_2_O_3_ (0001) substrates, which provide good lattice matching with PdTe, when in-plane rotation is taken into account (Figure S1, Supporting Information). The RHEED images in Figure 1a, monitored during the film growth, present the 6-fold in-plane symmetry and the alternating spacings of $\sqrt{3}$ *d*′ and *d*′ in two high-symmetry directions. The sharp streaks indicate the high structural quality of the film. The $\sqrt{3}$ *d*′ RHEED spacing in PdTe aligns well with the in-plane spacing, 2*d*, of Al_2_O_3_. Rutherford backscattering spectroscopy (RBS) shows a 1:1 ratio for Pd:Te, confirming the stoichiometry of the grown film (See Figure S2, Supporting Information).

**Figure 1:**
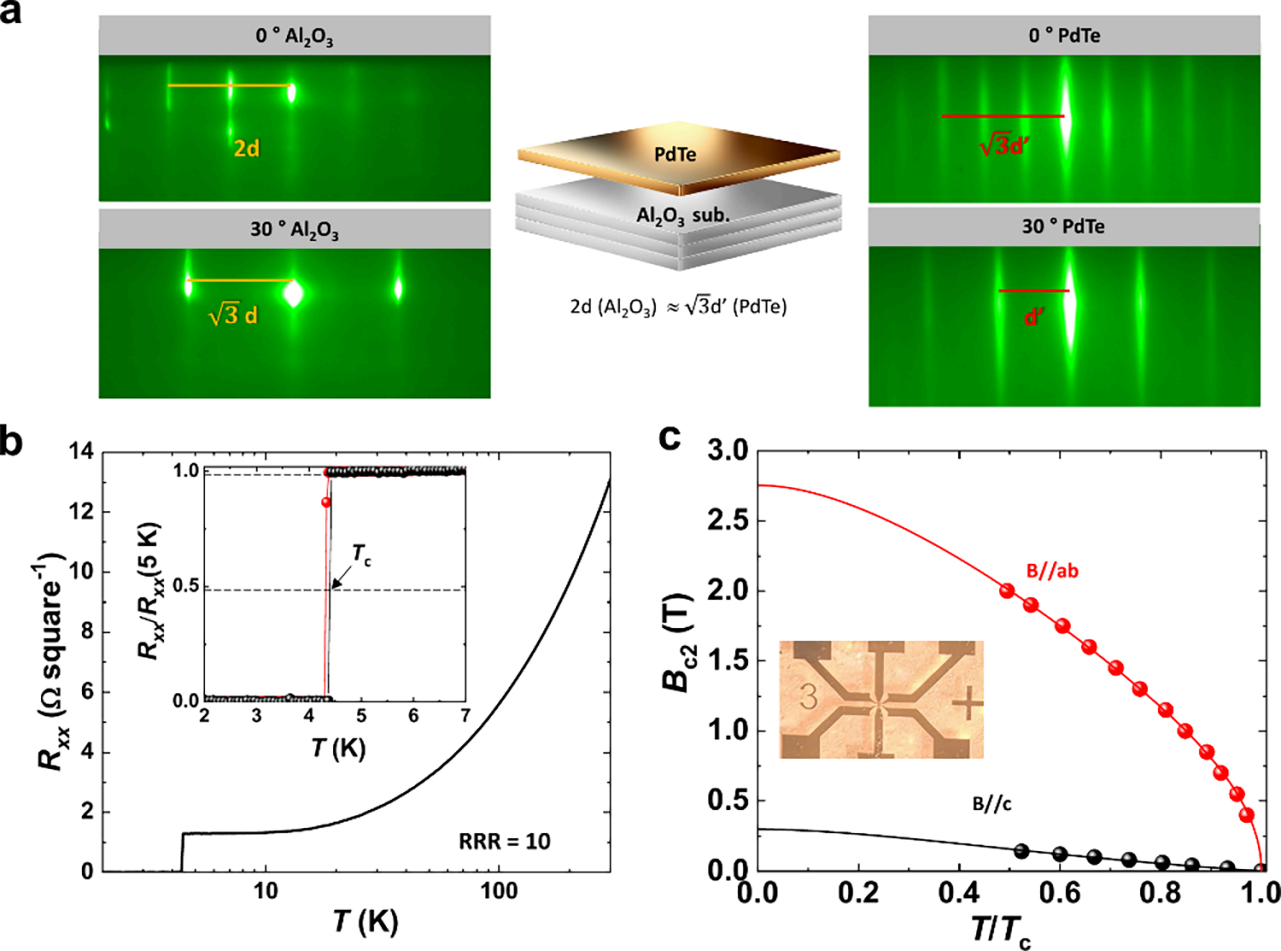
RHEED and transport properties of a PdTe film. (a) RHEED patterns along two high-symmetry directions for a 40 nm-thick PdTe film (right panels) and an Al_2_O_3_ substrate (left panels). A lattice match is observed between 2*d* and $\sqrt{3}$ *d*′ (see Figure S1, Supporting Information). (b) Semilog plot of the temperature-dependent longitudinal sheet resistance, *R*_*xx*_, of a PdTe film. The inset shows an enlarged graph of the normalized *R*_*xx*_ versus temperature, measured on a 10 × 10 mm^2^ sample using the van der Pauw geometry. The dashed line indicates half the normal state resistance. Red symbols and line represent the remeasured *R*_*xx*_(*T*) after 3 months of air exposure. (c) Upper critical fields measured along in- and out-of-plane directions. Symbols represent experiment data: black for out-of-plane and red for in-plane. Solid lines show fitted lines using the two-band WHH model. The inset displays the Hall bar-patterned sample used for the upper critical fields measurements. The channel area measures 25 μm in length and 30 μm in width.

Figure 1b exhibits the temperature-dependent longitudinal sheet resistance, *R*_*xx*_, of the PdTe film on a semilog plot. A sharp superconducting transition occurs at *T*_onset_ = 4.43 K with zero resistance temperature, *T*_0_ = 4.37 K, accompanied by a residual resistivity ratio, *RRR*, of 10, consistent with the reported *T*_c_ of bulk crystals.^14–17^ This sharp transition, *T*_onset_ – *T*_0_ = 0.06 K, combined with a high *RRR* of 10, indicates the high-quality epitaxial growth of the PdTe film. The inset displays the strong air stability of *T*_c_. We remeasured the PdTe thin film kept in air for 3 months (red symbols and line) and found that the degradation is minimal, *T*_c,as‑grown_ – *T*_c,aged_ < 0.1 K. This air stability is a strong advantage of PdTe compared to other nonoxide superconductors such as Fe­(Te,Se) for both research and applications. For instance, *T*_c_ of Fe-based superconductors can easily degrade even in a few days without a proper capping layer.^30^ We observed a large anisotropy of *B*_c2_ between out-of-plane and in-plane directions, as highlighted in Figure 1c. *B*_c2_ along the in-plane direction is 10 times larger than that along the out-of-plane direction. The high anisotropy, compared to the bulk value of 1.3, demonstrates that the thin film PdTe behaves like a 2D superconductor due to dimensional confinement.^13^ The *B*_c2_ data along the two different directions were fitted using the multiband Werthamer-Helfand-Hohenberg (WHH) model.^13,31^ This approach was necessary because the single-band WHH model, the standard approach for conventional superconductors, fails to fit *B*_c2_ in both directions simultaneously. This yielded a diffusivity ratio of η = D_2_/D_1_ = 0.21, where the *D*_1_ and *D*_2_ denote the diffusivities of each band. This indicates a strong asymmetry in quasiparticle transport between the two bands, consistent with the bulk case (See Figure S3, Supporting Information).^13^ Band structure calculations and magneto-transport studies on PdTe single crystals previously provided signatures of nontrivial band structure topology and the presence of two-electron and two-hole pockets in this system.^17^ The Hall effect measurements taken at 7 K on our PdTe thin films are also consistent with this previous study taken on the single crystals. he extracted mobilities are μ_h1_ = 434 cm^2^/V·s, μ_h2_ = 2,885 cm^2^/V·s, μ_e1_ = 343 cm^2^/V·s, and μ_e2_ = 2,573 cm^2^/V·s. Corresponding carrier densities are *n*_h1_ = 7.3 × 10^20^/cm^3^, *n*_h2_ = 2.6 × 10^19^/cm^3^, *n*_e1_ = 9.8 × 10^20^/cm^3^, and *n*_e2_ = 1.8 × 10^19^/cm^3^. (see Figure S4, Supporting Information).

Previously, thin film growth techniques such as MBE and sputtering led to only the PdTe_2_ phase. PdTe_2_ belongs to the CdI_2_-type crystal structure (P3̅m_1_), and PdTe belongs to NiAs-type (*P*6_3_/*mmc*). PdTe_2_ and PdTe share an identical in-plane crystal structure with slightly different lattice constants of 4.07 Å and 4.15 Å, respectively.^16,32^ One notable difference between them is that PdTe_2_ has a van der Waals bonding along the c-direction, whereas PdTe has a covalent/ionic bonding between Pd and Te. Moreover, PdTe_2_, being the end-member in the Pd-telluride phase diagram, can be readily grown with an adsorption-controlled self-limited growth mode. On the other hand, being surrounded by other competing phases, such as Pd_3_Te_2_ and Pd_2_Te_3_, it does not seem possible to grow PdTe in such a self-limited growth mode.

Not surprisingly, in our initial trials of direct growth of PdTe on Al_2_O_3_ substrate, we observed 3D spots with faint streaks in the RHEED patterns (not shown here), indicating poor crystallinity and mixed phases of the grown films. On the other hand, we were able to grow high-quality PdTe_2_ films under a Te-rich growth condition with minimal effort, so we decided to use PdTe_2_ as a buffer layer to grow PdTe on top. After preparing the highly crystallized PdTe_2_ thin film on the Al_2_O_3_ substrate, we deposited Pd and Te on the PdTe_2_ buffer layer with a nominal Te/Pd flux ratio of one. The RHEED spacing, *d*, showed an intermediate value between those of PdTe and PdTe_2_. The transport measurement showed a *T*_c_ of 2.7 K, which is between the bulk *T*_c_ of 4.5 K for PdTe and ∼1.8 K for PdTe_2_.^14,16,17^ This observation suggests that it is likely to be an intermediate or a mixed phase between PdTe and PdTe_2_.

To understand the growth mechanism and achieve high-quality PdTe films, we performed a systematic study on various Te/Pd ratios of *r,* as shown in Figure 2. After preparing a 32 nm-thick PdTe_2_ buffer layer, we deposited Pd and Te with the same amount of Pd used for the buffer layer but with different *r* values ranging from 3 to 0. Here, the *r*0 sample implies Pd growth without any Te supplied. Figure 2a shows that the RHEED spacings on the two high symmetry directions match well with those of PdTe_2_. With decreasing *r*, *d* shortens, indicating broadening of the in-plane lattice constant. With the *r* value reaching 0.25 (i.e., Te/Pd = 0.25), the lattice constant saturates at 4.14 Å. Interestingly, a third-order surface reconstruction peaks begin to emerge for *r* ≤ 0.25, which will be discussed later.

**Figure 2:**
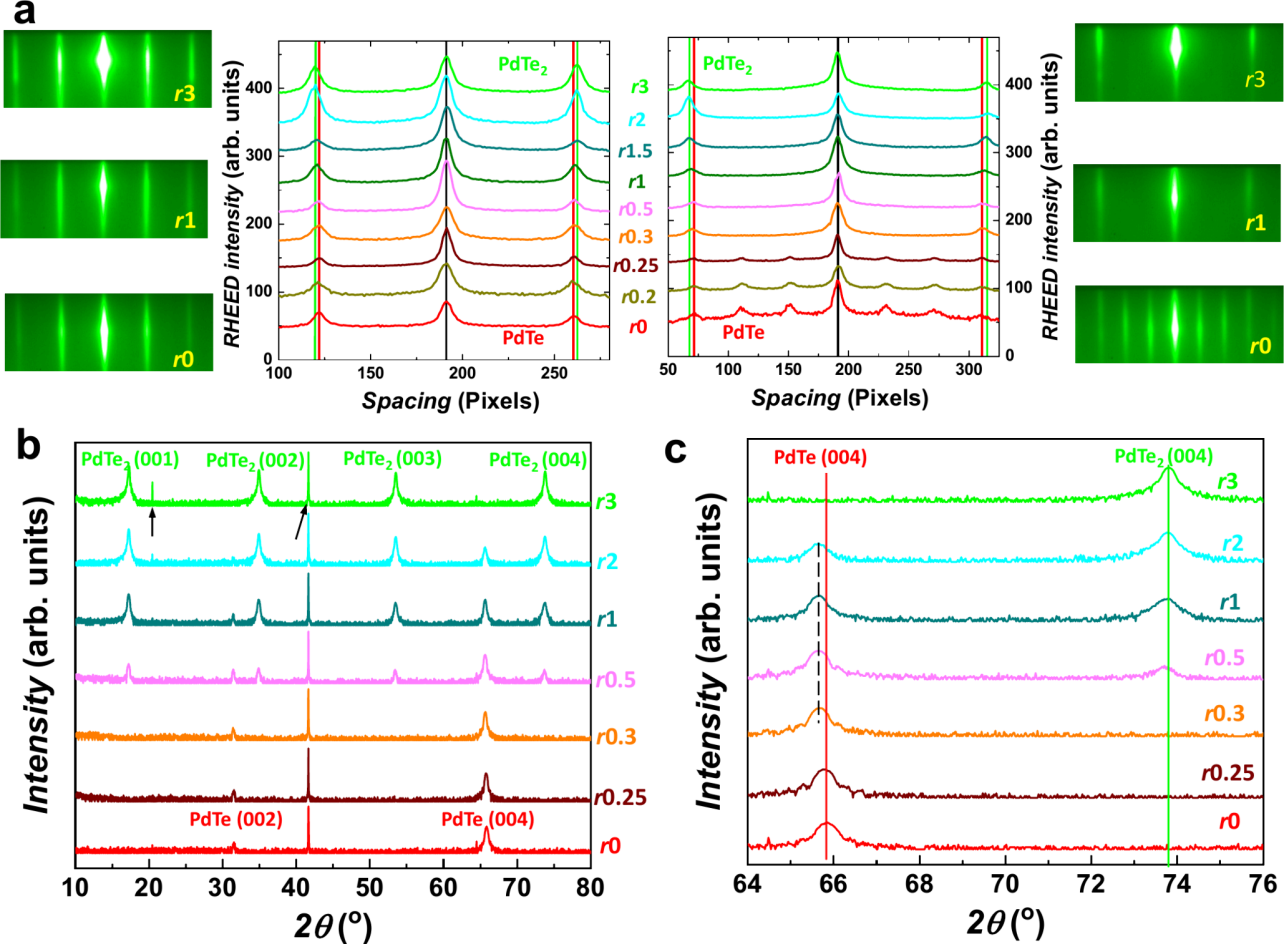
RHEED spaces and XRD analysis of Pd-telluride samples with varying *r* values. (a) As the *r-*value decreases, the RHEED spacing reduces and saturates at *r* = 0.25. At this point, 1/3-order surface reconstruction peaks begin to emerge. The surface reconstruction peaks work as a barometer, indicating the completion of the topotactic transformation. (b) Evolution of the (*00*n**) XRD peaks as a function of *r*-values. The black arrows indicate the (*00*n**) Al_2_O_3_ substrate peaks. (c) Enlarged view of the (004) peaks corresponding to PdTe and PdTe_2_. Red and green vertical lines indicate the PdTe and PdTe_2_ (004) peak angles. The dashed black line shifted to a smaller angle suggests an enlarged *c*-axis lattice constant of PdTe in samples with intermediate *r* values.

The XRD patterns in Figure 2b also exhibit a phase transformation from PdTe_2_ to PdTe with decreasing *r*. All of the XRD peaks in the *r*3 sample can be identified as PdTe_2_ (00*n*) and Al_2_O_3_ (00*n*) peaks. As *r* decreases, PdTe­(00*n*) peaks appear and coexist with the PdTe_2_(00*n*) peaks. When *r* reaches 0.3, the PdTe_2_(00*n*) peaks vanish entirely, and only PdTe­(00*n*) peaks remain. However, upon closer inspection of the XRD data (Figure 2c), the PdTe(004) peak appears at a slightly lower angle than the known bulk value, as indicated by the dashed line in Figure 2c. This could be due to the presence of some interstitial Te species, and as the Te flux is further reduced to *r* = 0.25, this peak eventually shifts to a bulk value.

The normalized longitudinal resistance as a function of temperature, shown in Figure 3a, reveals how the superconducting transition temperature evolves as the film transforms from PdTe_2_ to PdTe. Except for the *r*3 sample, which is shown above to be PdTe_2_ (*T*_c_ ≈ ∼1.8 K), all the other samples exhibit *T*_c_ above 1.9 K. Figure 3b summarizes the data for all the samples. Bulk-like *T*_c_ (4.4 K) of the PdTe phase is observed for *r* ≤ 0.25, but as *r* increases from 0.25 to 0.3, *T*_c_ abruptly drops to 2.7 K, and *RRR* also drops sharply, reaching a minimum at *r*0.5. With a further increase in *r*, *RRR* rebounds and reaches 15 at the *r*3 sample (See Figure S5, Supporting Information, for further details). The sudden drop in *RRR* between *r*0.25 and *r*0.3 is likely due to the development of atomic-scale disorder, such as the interstitial Te in the PdTe phase, as suggested above based on the XRD data in Figure 2.

**Figure 3:**
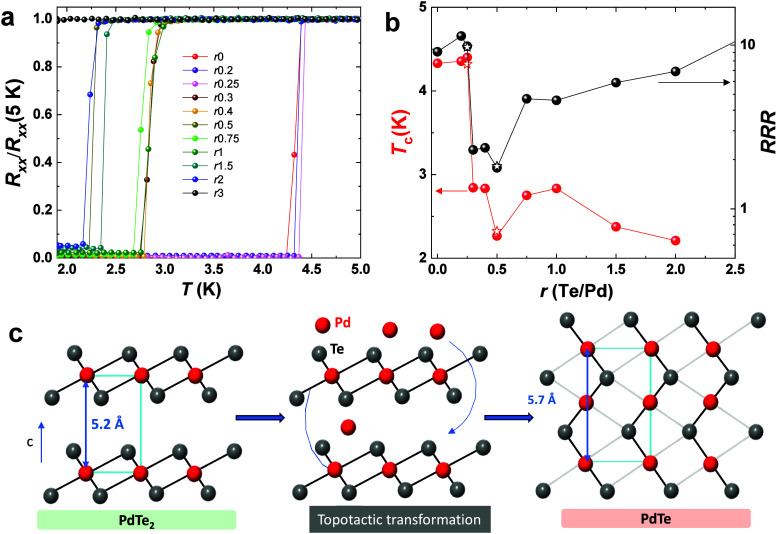
Transport properties of Pd-telluride films with varying *r* values and a schematic of the topotactic transformation. (a) Normalized *R*_*xx*_ as a function of temperature for the samples as *r* varies. (b) Summary of *T*_c_ and *RRR* for the measured samples. Star symbols represent another sample prepared under identical conditions, demonstrating the reproducibility of the synthesis method. (c) A schematic of the topotactic transformation from PdTe_2_ to PdTe, driven by the diffusion of excess Pd ions in samples with *r* < 1.

These observations strongly suggest that by growing Pd under Te-poor growth conditions on top of PdTe_2_, the entire film, including the PdTe_2_ buffer layer, transforms into the PdTe phase. This implies that Pd can readily diffuse through the film at the growth temperature, as visualized in the schematic of Figure 3c. Under low *r* conditions, excess Pd atoms diffuse into the PdTe_2_ buffer layer and bond with neighboring Te atoms within the van der Waals gap, converting PdTe_2_ into PdTe. Such a process is generally referred to as a topotactic transformation. Even if growth begins with the PdTe_2_ buffer, high Pd diffusivity facilitates the topotactic transformation to convert PdTe_2_ into a high-quality PdTe film for *r* ≤ 0.25. Interestingly, the surface reconstruction peaks observed in the RHEED of *r*0.25 samples serve as a barometer indicating that the film has been completely transformed into PdTe. Notably, the *r*0 sample exhibits a slightly lower *T*_c_ and *RRR* compared to the *r*0.25 sample. This could be due to the presence of Pd clusters on the surface of the *r*0 sample, as confirmed by Atomic Force Microscope (AFM) images in Figure S6 (Supporting Information).

In Figure 3, it is notable that while *T*_c_ and *RRR* correlate with each other for *r* < 0.5, such is not the case for *r* > 0.5. This is because on the *r* > 0.5 side, even though the reduction in disorder (i.e., increase in RRR) helps enhance the superconductivity, the *T*_c_ should eventually drop toward that (1.8 K) of PdTe_2_.

To further investigate how the topotactic transformation occurs, we performed STEM studies on three representative samples: *r*3, *r*0.5, and *r*0.25. As shown in Figure 4, the STEM images of the *r*0.25 and *r*3 samples display the NiAs-type (PdTe) and CdI_2_-type (PdTe_2_) stacking patterns, respectively. A distinct van der Waals gap is clearly visible in the *r*3 sample. The measured lattice constants along the *c*-axis are 5.7 Å for *r*0.25 and 5.2 Å for *r*3, consistent with the known values of PdTe and PdTe_2_.^15,17,32^ In contrast to the homogeneous structures observed in the *r*3 and *r*0.25 samples, the *r*0.5 sample displays mixed stacking patterns in Figure 4b, which reveals a clear boundary between the two phases, PdTe (top) and PdTe_2_ (bottom). The fact that we observe a sharp superconducting transition in all these films at temperatures between that of PdTe (4.4 K) and PdTe_2_ (1.8 K) suggests that the PdTe/PdTe_2_ heterostructures form transparent interfaces with strong proximity coupling. However, a closer inspection of STEM images near the interface between the PdTe and PdTe_2_ phases reveals that the detailed structures in each region deviate from those of the pure PdTe and PdTe_2_ phases. The stacking angle varies from ∼47° to ∼50° in PdTe and from ∼54° to ∼52° in PdTe_2_ near the interface, corresponding to local out-of-plane strains of ∼4.6% tensile for PdTe and ∼2.6% compressive for PdTe_2_, highly localized at the PdTe/PdTe_2_ interface.

**Figure 4:**
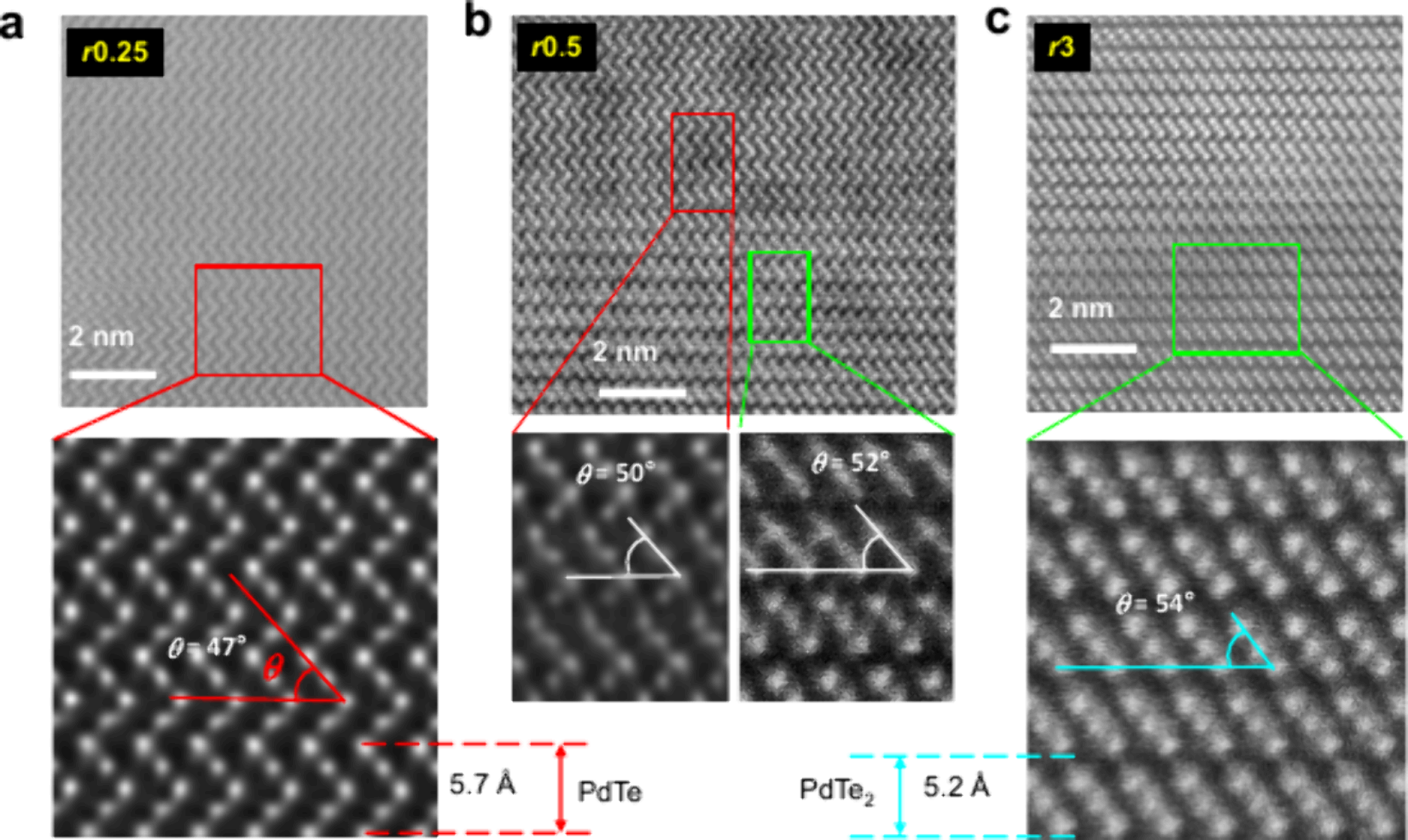
STEM images of Pd-telluride films with varying *r* values. (a) *r* = 0.25 sample shows the PdTe structure with a stacking angle of 47°. (b) *r* = 0.5 sample exhibits two distinct regions: the upper region displays a PdTe-like stacking with an angle of 50°, while the lower region shows a PdTe_2_-like stacking with an angle of 52°, presumably due to some compositional mixing between the two phases. (c) *r* = 3 sample presents a stacking angle of 54°, characteristic of PdTe_2_.

## Conclusions

In conclusion, we have developed the first high-quality, superconducting PdTe thin films. This was made possible by depositing Pd in a Te-poor growth condition on a PdTe_2_ buffer layer through a topotactic transformation process. The optimized films exhibited a bulk-like superconducting transition with a narrow transition width of 0.06 K and a high *RRR* of ∼10. Comprehensive structural analyses, including XRD, RBS, AFM, and RHEED, revealed a clear phase evolution between PdTe_2_ and PdTe as a function of the Te/Pd ratio. Interestingly, over the entire flux range, only PdTe_2_, PdTe, and their mixtures—without any other second phases such as Pd_2_Te_3_ or Pd_3_Te_2_—were observed in the films. With the high-quality PdTe films available, it is now feasible to probe previously inaccessible topological properties, such as Majorana zero modes, in this novel topological superconductor candidate. Moreover, the 2D superconducting behavior and exceptional air stability make this system a promising platform for topological or magnetic superconductor heterostructures, thereby facilitating the realization of proximity-induced TSCs.

## Materials and Methods

### Thin-Film Growth

We prepared PdTe films on 10 × 10 × 0.5 mm^3^ Al_2_O_3_ (0001) using a custom-built MBE system (SVTA) with a base pressure of ∼10^–10^ Torr. Al_2_O_3_ substrates were cleaned ex-situ by UV-generated ozone and in situ by heating to 750 °C under oxygen pressure of 5 × 10^–7^ Torr. Before depositing the PdTe, we employed a PdTe_2_ buffer layer. PdTe_2_ buffer layer and PdTe thin film were grown at 275 and 250 °C, respectively. High-purities of Pd and Te were thermally evaporated using Knudsen diffusion cells for film growth. All source fluxes were calibrated in situ using a quartz crystal microbalance.

### Transport Measurements

All transport measurements were performed using the van der Pauw geometry, except for upper critical fields measurements. For upper critical fields measurements, a Hall bar pattern was prepared using optical lithography with a channel length of 25 μm and a width of 30 μm. Keithley 2400 source-measure units and 7001 switch matrix system were controlled by a LabView program for longitudinal and Hall resistance measurements. A Physical Property Measurement System (PPMS, Quantum Design Inc.) was employed to cool down to 2 K.

### HAADF-STEM and XRD Measurements

High-angle annular dark-field scanning transmission electron microscopy (HAADF-STEM) characterizations were performed at the Instrumentation Center for Physical Science in China. TEM samples were prepared by a focused ion beam (Helios G5, Thermo Fisher Scientific) using 2.0 keV Ga^+^ ions for the final milling. HAADF-STEM images were acquired with 300 keV electrons with collection angles ranging from 67 to 275 mrad using a double aberration-corrected JEOL.

## Supplementary Material



## Data Availability

All data are available in the manuscript or the Supporting Information and are available from the corresponding author upon request.

## Notes

Schematic of the commensurate relationship between Al_2_O_3_ and PdTe; an RBS spectrum; two-band WHH model fits of the upper critical fields; a multiband fit of the Hall measurement at the normal state; temperature-dependent resistance curves with varying *r* values; AFM images; and a low-magnification STEM image of PdTe films (PDF)
