# Supplementary material for: Superconducting PdTe Thin Film via Topotactic Transformation, toward Topological Superconductors
Source: ACS Appl Nano Mater. 2026 May 29;9(23):10684–90. doi: 10.1021/acsanm.6c00907 (PMC13270465; doi:10.1021/acsanm.6c00907)
Supplement: Supplementary file 1 [file an6c00907_si_001.pdf]

Supporting information

Superconducting PdTe Thin Film Via Topotactic Transformation, Toward Topological Superconductors

Hee Taek Yi[a, *], Min Ge[b], Renjie Xie[c], Colby J. Stoddard[a], David H. Yi[d], Xiaoyu Yuan[a], Xiong Yao[c], and Seongshik Oh[a, *]

[a] *Department of Physics & Astronomy, Rutgers University, Piscataway, New Jersey 08854, USA*
[b] *The Instruments Center for Physical Science, University of Science and Technology of China, Hefei 230026, China*
[c] *Ningbo Institute of Materials Technology and Engineering, Chinese Academy of Sciences, Ningbo 315201, China*
[d] *Department of Physics, Mellon College of Science, Carnegie Mellon University, Pittsburgh, Pennsylvania 15213, USA*

*E-mail: taeggy@physics.rutgers.edu and ohsean@physics.rutgers.edu

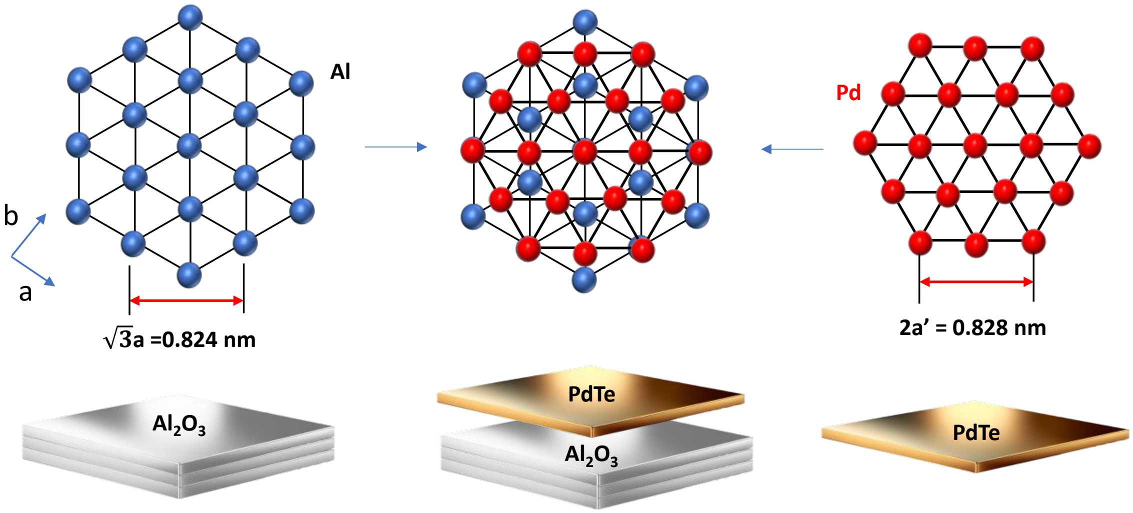

Figure *S*1. Schematic illustration of rotational commensurate lattice matching. Blue and red symbols represent Al and Pd atoms, respectively. The illustration highlights the commensurate relationship between the in-plane lattice constant of Al_2O_3 and that of PdTe.

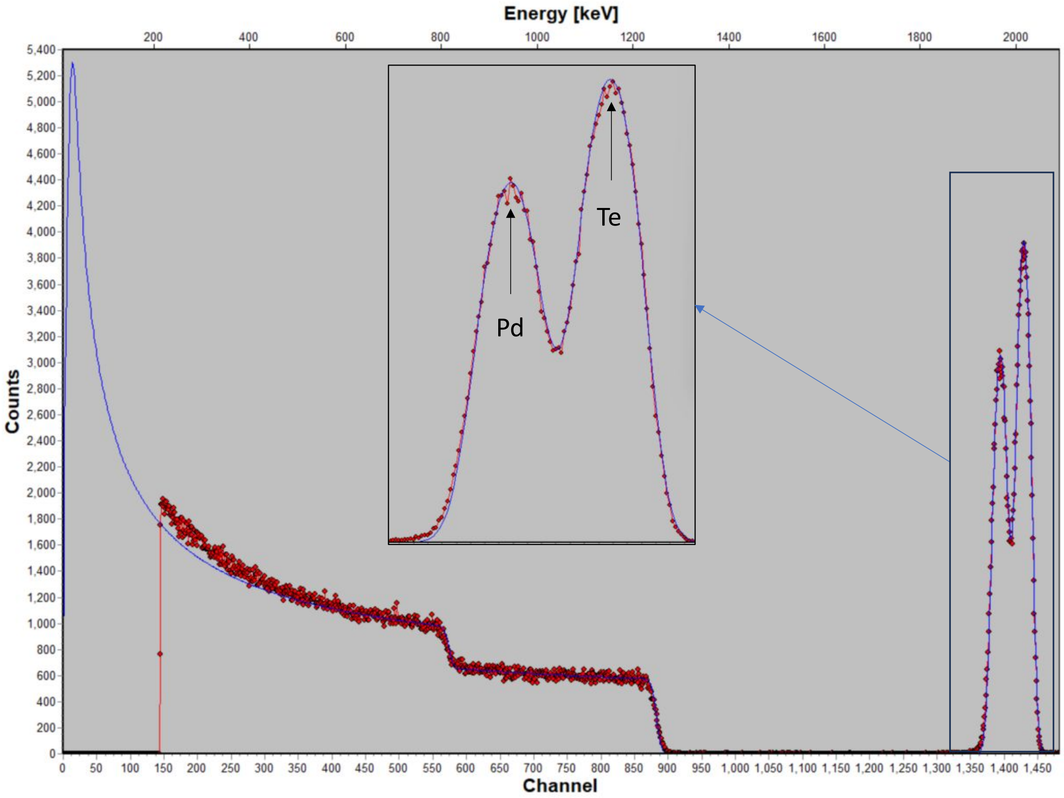

Figure *S*2. Rutherford backscattering spectroscopy (RBS) spectrum recorded on a PdTe thin film. RBS experimental data for the *r*0.25 PdTe film (red circles) are compared with simulated data (blue line). The inset shows an enlarged view of the spectrum, highlighting the Pd and Te signals. The simulation corresponds to a stoichiometric composition of PdTe with a 1:1 ratio.

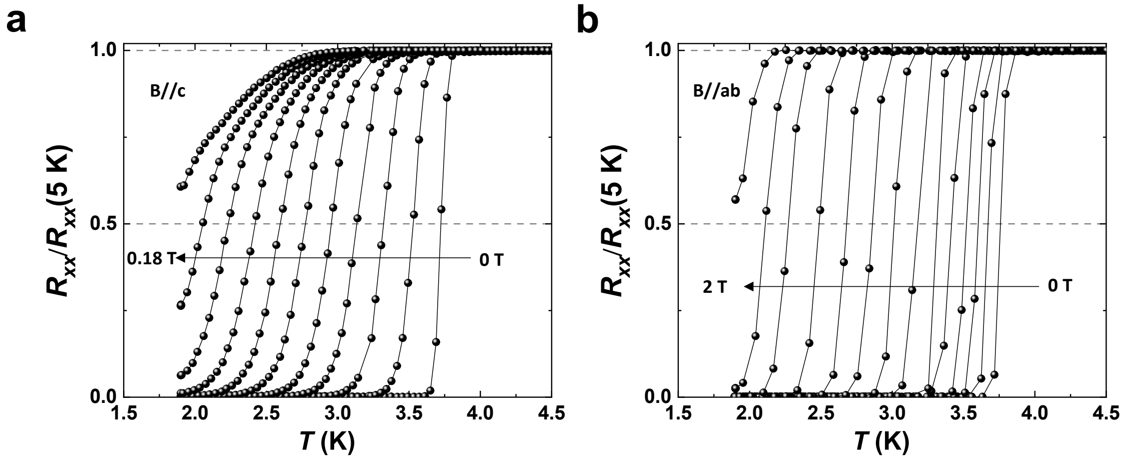

Figure *S*3. Temperature-dependent R_{xx} of the PdTe thin film under varying magnetic fields along (a) perpendicular and (b) parallel direction to the ab plane.

The upper critical field is fit to the two-band Werthamer-Helfand-Hohenberg (WHH) model defined by

$$\frac{w}{\lambda_0}\big(\ln t + U(h)\big)\big(\ln t + U(\eta H)\big) + (1+\lambda_-/\lambda_0)\big(\ln t + U(h)\big) + (1-\lambda_-/\lambda_0)\big(\ln t + U(\eta h)\big) \qquad [Eq.1]$$

where $U(x) = \psi\left(x+\frac{1}{2}\right) - \psi\left(\frac{1}{2}\right)$, $t = T/T_c$, $\eta = D_2/D_1$, $w = \lambda_{11}\lambda_{22} - \lambda_{12}\lambda_{21}$, $\lambda_- = \lambda_{11} - \lambda_{22}$, $\lambda_0 = \sqrt{\lambda_-^2 - 4\lambda_{12}\lambda_{21}}$, and $\psi(x)$ is the digamma function.[1] The parameters D_1 and D_2 are the intraband diffusivities of bands 1 and 2, respectively. The constants λ_{11} and λ_{22} quantify the intraband superconducting coupling, while λ_{12} and λ_{21} quantify the interband coupling. For thin films, [2,3]

$$h = \frac{D_1}{4\pi kT}\left(2eH_{c,2}\,\sin\theta + \frac{1}{3\hbar}\left(deH_{c,2}\,\cos\theta\right)^2\right)[Eq.\,2]$$

where $H_{c,2}$ is the upper critical field, $-e$ is the electron charge, k is Boltzmann's constant, d is the effective superconducting film thickness and θ is the angle from the film plane. We used the coupling constants for single-crystal PdTe found in Ref.[4] and found the best fit for η, D_1, and d. Note that λ_{12} and λ_{21} only appear in Eq. 1 as the product $\lambda_{12}\lambda_{21}$, so we only considered the product $\lambda_{12}\lambda_{21}$. Table S1 shows the fit parameters for a simultaneous fit of the in-plane and out-of-plane upper critical fields.

$\sqrt{\lambda_{12}\lambda_{21}}$	$\lambda_{22}/\lambda_{11}$	η	D_1 (m^2/s)	d (nm)
0.627	1.32	0.21	3.66×10^{-3}	12.5

Table *S*1. Fit parameters to Eq. 1 for simultaneous fit to in-plane and out-of-plane upper critical fields.

The longitudinal and transverse conductance converted from the measured longitudinal and transverse resistances with the channel ratio, l/w =1.2 are defined as follows:

$$\sigma_{xx} = \frac{\rho_{xx}}{\rho_{xx}^2+\rho_{xy}^2} \text{ and } \sigma_{xy} = \frac{-\rho_{xy}}{\rho_{xx}^2+\rho_{xy}^2} \text{ with } \rho_{xx} = \frac{R_{xx}}{1.2} \text{ and } \rho_{xy} = R_{xy}$$

The conductivity of an N-carrier system is given by[5]

$$\sigma_{xx} = \sum_{i=1}^{N} \frac{n_i q_i \mu_i}{1+\mu_i^2 B^2} \text{ and } \sigma_{xy} = \sum_{i=1}^{N} \frac{s_i n_i q_i \mu_i^2 B}{1+\mu_i^2 B^2}$$

where B is the applied magnetic field and n_i,q_i, and μ_i are the carrier density, charge, and mobility of the ith carrier, respectively. The coefficient s_i is -1 for electrons and $+1$ for holes. We performed a simultaneous least-squares fit to σ_{xx} and σ_{xy} to determine the density, mobility, and type of each carrier. The two- and three-carrier models yielded poor fits to the experimental data, whereas the four-carrier model provided an excellent fit as shown in Fig. *S*4. The extracted mobilities are μ_{h1} = 434 cm^2/V·s, μ_{h2} = 2,885 cm^2/V·s, μ_{e1} = 343 cm^2/V·s, and μ_{e2} = 2,573 cm^2/V·s. Corresponding carrier densities are n_{h1} = 7.3 × 10^{20}/cm^3, n_{h2} = 2.6 × 10^{19}/cm^3, n_{e1} = 9.8 × 10^{20}/cm^3, and n_{e2} = 1.8 × 10^{19}/cm^3.

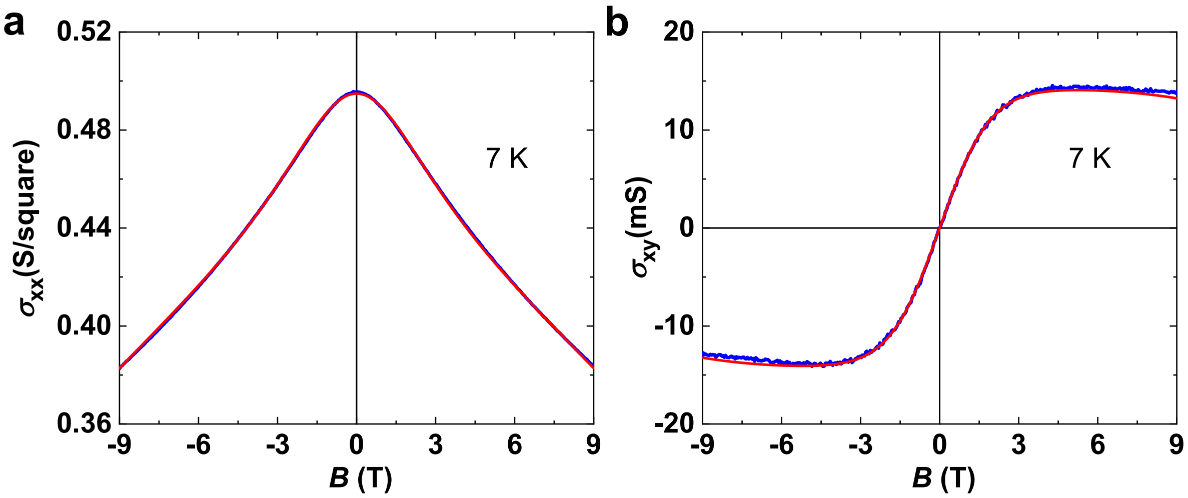

Figure *S*4. Hall effect measurement at 7 K in the normal state (a) longitudinal and (b) Hall conductance as a function of applied magnetic fields. Black and red curves represent the experimental and the fitted results, respectively.

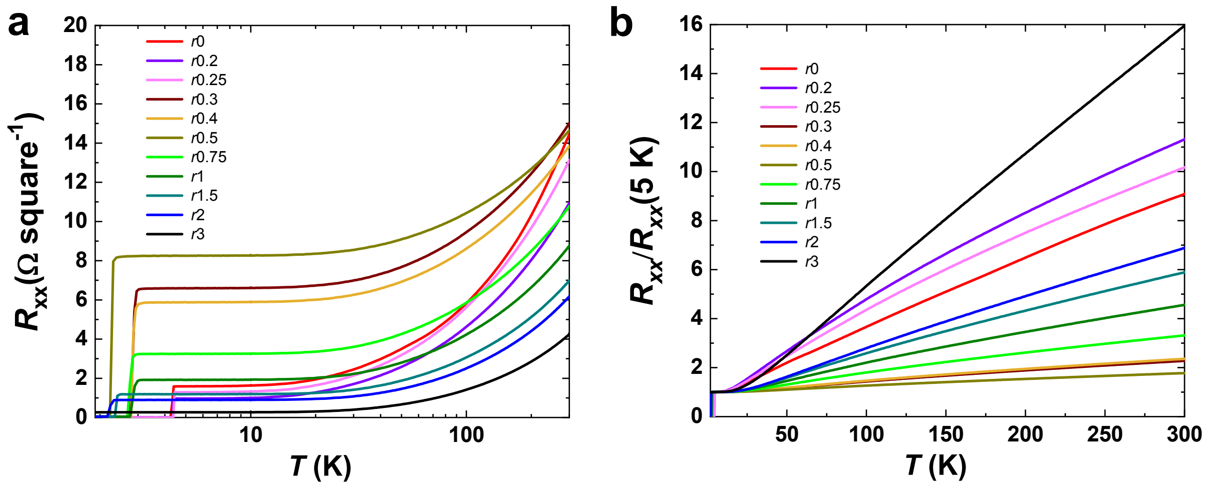

Figure *S*5. Temperature-dependent resistance of Pd-telluride films with varying r values. (a) R_{xx} as a function of temperature for samples with various r values. (b) normalized resistance versus temperature, highlighting *RRR* across the samples.

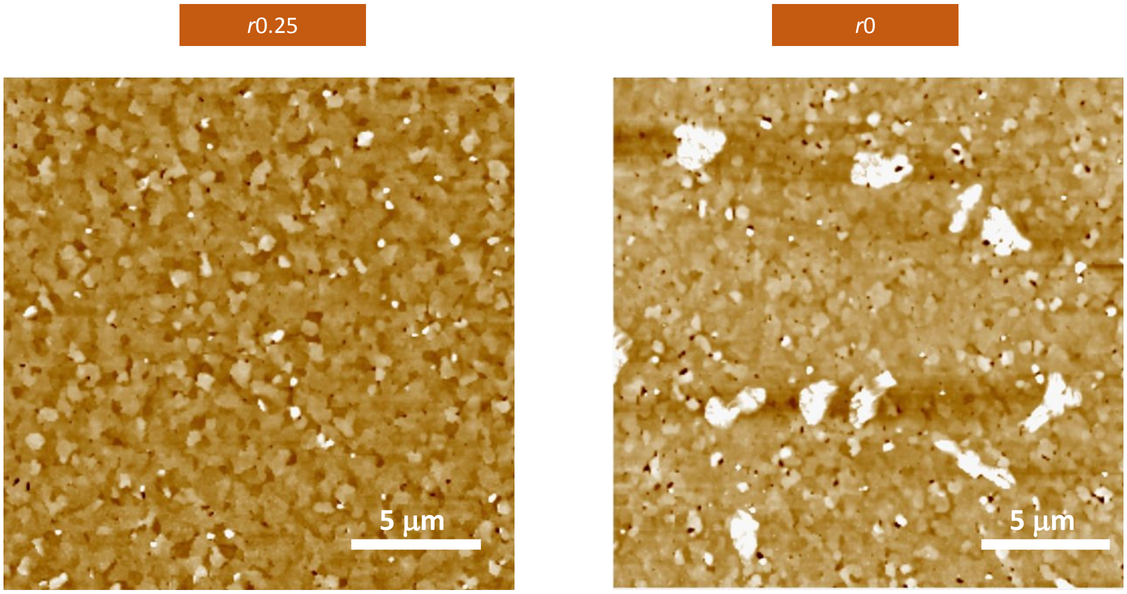

Figure *S*6. Atomic Force Microscopy (AFM) images of the surface for *r*0.25 and *r*0 samples with a scan size of 20 x 20 µm^2. The AFM image for the *r*0 sample displays clustered islands of excess Pd atoms.

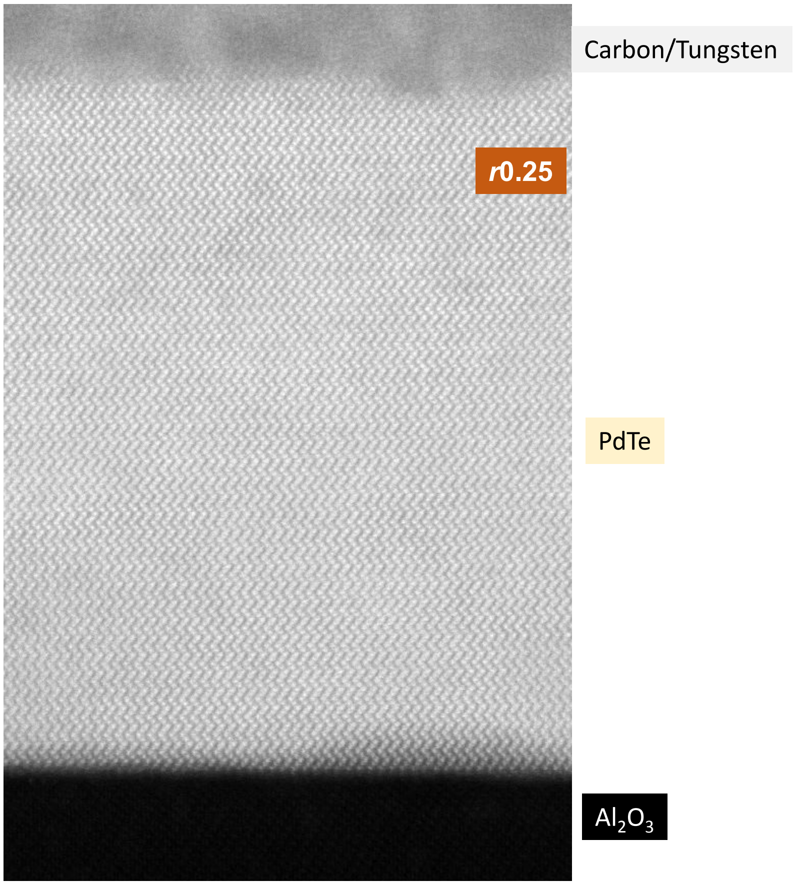

Figure *S*7. STEM image of Pd-telluride film with $r = 0.25$ sample. The low-magnification cross-sectional image shows a zigzag pattern across the entire film, with a clear interface between PdTe and the Al_2O_3 substrate.

References

[1] A. Gurevich, *Phys. Rev. B* **2023**, 67, 184515.
[2] A. Gurevich, *Physica C* **2007**, 456, 160.
[3] A. Koya, *Phys. Rev. B* **1973**, 7, 5544.
[4] V. Amit, *et. al.*, arXiv:2408.06424.
[5] J. Lindemuth, Hall Effect Measurement Handbook, Westerville, OH: Lake Shore Cryotronics, **2020**.
